# Traveling together! - Intrathymic thyroid tissue in a patient with Graves’ disease

**DOI:** 10.4322/acr.2024.506

**Published:** 2024-07-30

**Authors:** Hugh Ellis McCormick, Sidrah Khawar, Ameer Hamza

**Affiliations:** 1 University of Kansas Medical Center, Department of Pathology and Laboratory Medicine, Kansas City, Kansas, USA

**Keywords:** Ectopic thyroid, Intrathymic thyroid, Thoracic intrathymic thyroid, Benign thymic nodule, Mediastinal mass, Graves’ disease

## Abstract

Ectopic thyroid tissue is rare in the general population and more prevalent in people who have existing thyroid disease. Common anatomical sites of ectopic thyroid tissue include the lateral cervical region, thyroglossal duct, mediastinum, lingual, sublingual, and submandibular region. Intrathymic ectopic thyroid tissue is exceedingly rare. The purpose of this report is to describe one such case in a 52-year-old African-American female with Graves’ disease. The patient presented for a physical exam and follow-up. During the exam, an incidental mediastinal mass was discovered, which was evaluated by imaging studies and subsequently was resected. Histologically, the mass was composed of variable-sized thyroid follicles lined by a monolayer of cuboidal to columnar follicular epithelial cells and filled with eosinophilic colloid, surrounded by a rim of unremarkable compressed thymic tissue.

## INTRODUCTION

Ectopic thyroid tissue is found in 1/100,000-300,000 of the population globally and in approximately 1/4,000-8,000 of people with existing thyroid disease.^1^ Ectopic thyroid tissue is usually discovered in neonates but may present later in the fourth to sixth decades of life as well.^1^ The etiology behind the higher prevalence of ectopic thyroid tissue in patients with thyroid disease is unclear. Still it may either be due to more robust screening procedures or the possibility of a separate pathogenic correlation that is not yet understood.^1^ Common anatomical sites of ectopic thyroid tissue include lateral cervical region, thyroglossal duct, mediastinum, lingual, sublingual, and submandibular region.^1^ And while ectopic thyroid tissue is itself a rare phenomenon, the chances of ectopic tissue presenting intrathymically is exceedingly rare. The cause of this is uncertain but hypothesized to be due to a similar pathway of descent of the thymus and the thyroid during development.^2^ It was first described in the literature in 1994 as a novel finding termed “thoracic intrathymic thyroid”. Ever since, less than 10 cases have been reported in the English medical literature. Herein, we will describe a case of intrathymic thyroid tissue as it is imperative to document these cases in medical literature along with their clinical findings and diagnostic features.

## CASE REPORT

**Figure 1:**
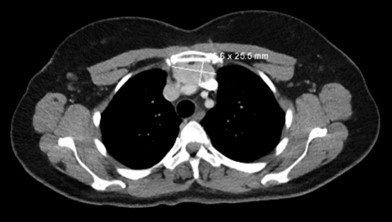
Thoracic CT scan demonstrating a thymic mass.

A 52-year-old African American female with a history of Grave’s disease and multinodular goiter presented for a physical exam and follow-up. Here most recent thyroid ultrasound was from a couple of years ago that showed diffuse heterogeneity and increased hypervascularity of the gland, multiple subcentimeter nodules throughout both lobes with largest 7 mm. She never had a fine needle aspiration cytology performed. During the exam, an incidental mediastinal mass was discovered, which was evaluated by a series of CT and PET scans. Imaging revealed a low-level FDG uptake suggestive of thymoma with a differential of lymphoproliferative disorder or lymph node metastasis. The thyroid gland was mildly enlarged and slightly heterogeneous, without a mass or dominant nodule. There were no enlarged mediastinal, hilar, or axillary lymph nodes. No diagnosis of a primary tumor had been made; however, with the patient’s history of Grave’s disease and diffuse goiter, an occult primary tumor could be possible. Follow-up imaging showed a stable mediastinal mass measuring 4.6 x 2.6 cm (Figure 1).

**Figure 2:**
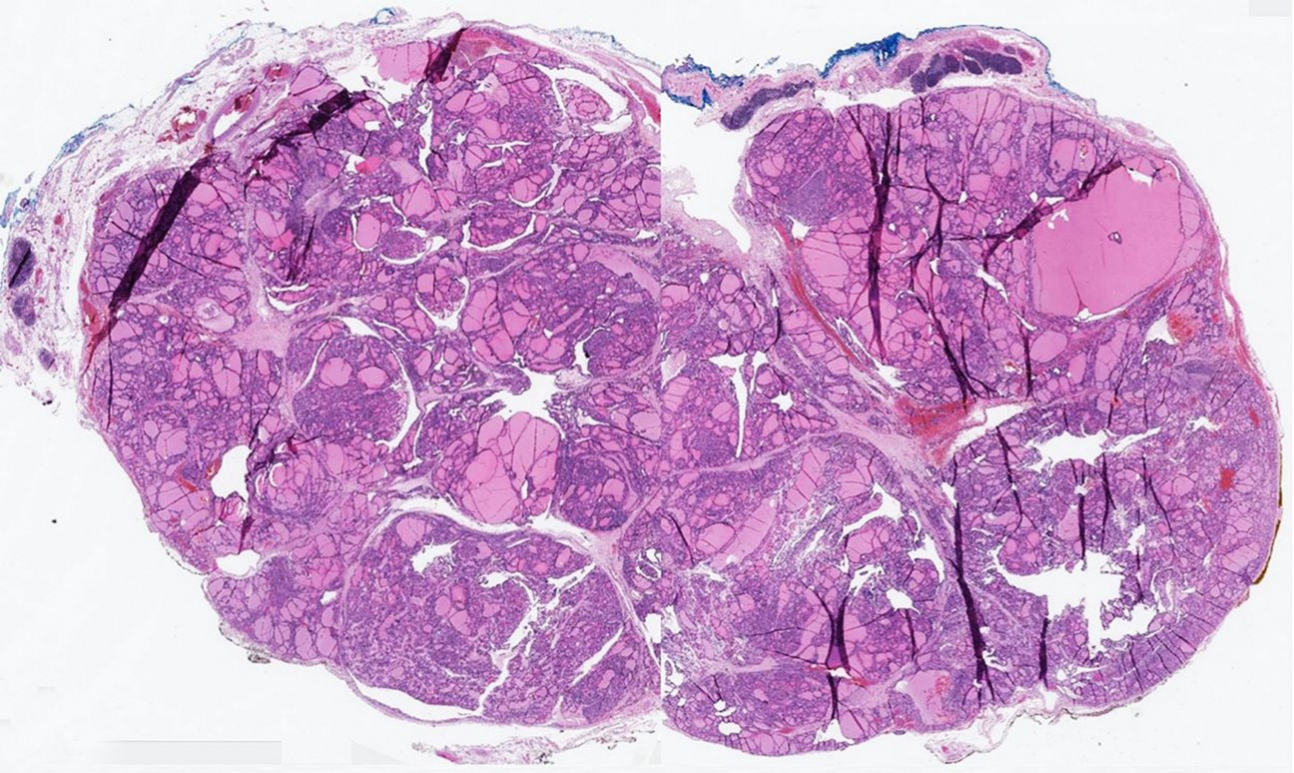
Photomicrographs of the surgical specimen. – Wholemount image showing the heterotopic thyroid tissue and surrounding unremarkable thymic tissue (H&E 5x).

**Figure 3:**
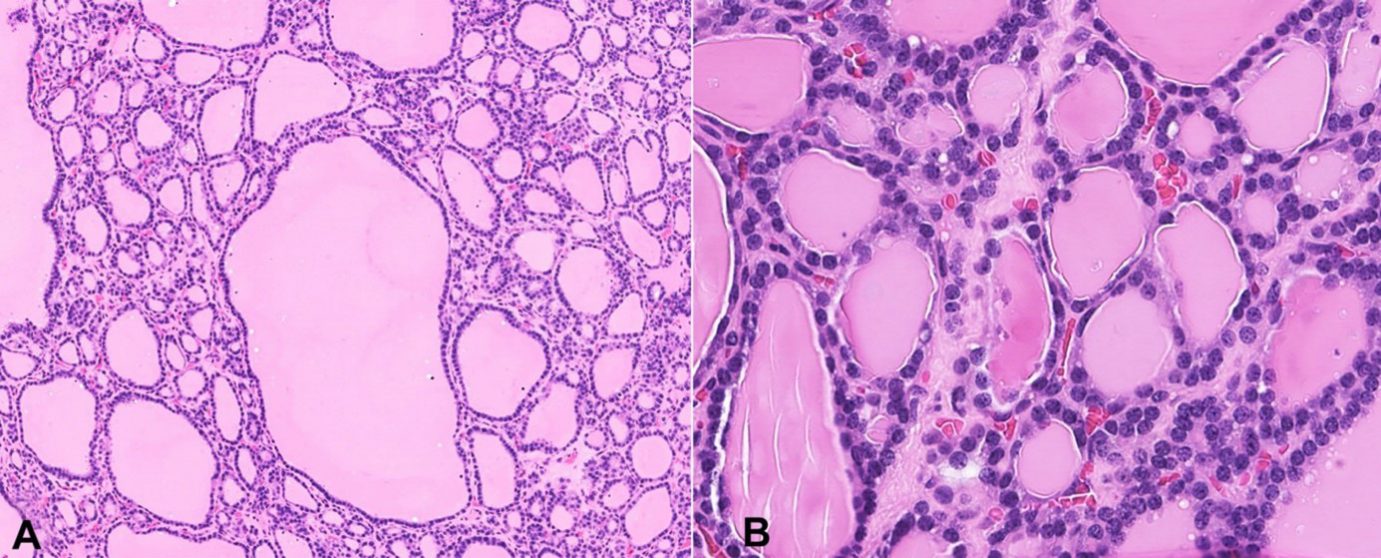
Medium (**A**) and high (**B**) power photomicrographs showing the cytologic details of the thyroid tissue (H&E 100x and 400x, respectively).

The patient subsequently underwent a total thymectomy. Prior to surgery the patients TSH was 0.49 (Normal range 0.35 – 5.00 µU/mL). During surgery, a tissue plane was noted between the mass and the innominate vein. The mass abutted the left pericardium / phrenic nerve without apparent invasion. There was also no invasion of other mediastinal structures. The tissue was sent to pathology for analysis. Grossly, the left half of the thymus measured 14 x 4.8 x 2.2 cm and weighed 44g. The right half of the thymus was 10.7 x 2.6 x 0.9 cm and weighed 11g. Sectioning revealed a 5.6 x 4.7 x 2.2 cm tan-brown, focally cystic, lobulated, and well-circumscribed mass within the left half of the thymus (Figure 2). No distinct lesions were seen on the right side. Microscopically, the sections from the mass showed benign thyroid tissue, composed of variable-sized thyroid follicles lined by a monolayer of cuboidal to columnar follicular epithelial cells and filled with eosinophilic colloid (Figure 3A and 3B). The surrounding thymic tissue was compressed but was otherwise unremarkable (Figure 2). The final diagnosis of heterotopic intrathymic multinodular benign thyroid tissue was rendered.

The patient’s post-operative recovery was uneventful, and her Grave’s disease remains stable. One month after surgery her TSH level was 1.06 (Normal range 0.35 – 5.00 MCU/ML); T4-free was 1.24 (Normal range: 0.58 - 1.64 ng/dL) and T3 total was 106 (Normal range: 80 - 200 ng/dL). Thyroid receptor antibodies were not tested post-surgery.

## DISCUSSION

In humans, the development of the thyroid gland begins approximately 20-24 days after fertilization, arising caudal to the tuberculum impar, which is also known as the median tongue bud.^3^ As the embryo and tongue grow, the developing thyroid gland descends into the neck and is positioned in its final anatomic location mid-neck by six to eight weeks.^3^ Additionally, the thymus follows a similar and proximal pattern of descent in fetal development as the thyroid.^4^ Because the descent of the thyroid and thymus are so close, there is a possibility of the thymus to adhere to the thyroid when the thymus passes the thyroid during their descent. Therefore, it is common for the thyroid tissue to be present at heterotopic sites, ranging from the tongue to the thoracic organs and including the thymic tissue remnants in the neck and mediastinum.^5^ As noted, it is an infrequent finding, but there also is a good possibility that this may be occurring more commonly than documented, as it is predominantly asymptomatic and may be going without discovery and documentation. Of note, there are occasional reports of presenting complaints such as mediastinal and chest pain.^2,6^

It is pertinent to note that this is not a case of substernal goiter or intrathyroidal thymic tissue; that is much more common incidental finding, perhaps due to more thyroid surgeries compared to thymic surgeries.^7,8^ Ectopic thyroid tissue is most commonly found incidentally in routine imaging or physical for other conditions or preoperative imaging and, again may be found anywhere along the line of the obliterated thyroglossal duct, usually from the tongue to the diaphragm.^1,3^

In symptomatic patients, the presenting complaint depends on the location of the ectopic thyroid tissue. In thymic / mediastinal location, most patients are asymptomatic as in the case of our patient. Two patients described in the literature presented with substernal chest pain.^2,6^ Another patient was discovered on mesothelioma screening exam and X-ray following long-term asbestos exposure.^2^ Two patients had concurrent malignancies and concern for metastatic disease.^5,9^

A thymic / mediastinal mass or nodule is the common radiologic description of the intrathymic thyroid tissue.^2^ When available, differential diagnoses based on imaging included thymoma or metastatic malignant process, as in our case. Table 1 summarizes the clinicopathologic features of ectopic intrathymic thyroid tissue cases in the existing literature. All patients were treated surgically with resection.

**Table 1:** Clinicopathologic features of intrathymic thyroid tissue cases reported in the literature

Ref	Age / Gender	Presenting Complaint	Physical Exam	Imaging	Pathology
^2^	61	None	Normal	Anterior mediastinal mass on CT scan	Grossly large mass within the body of the thymus without evidence of invasion. Tissue samples consisted of thyroid tissue closely associated with atrophic thymic tissue.
^2^	41	Substernal chest pain	Normal	Anterior mediastinal mass on CT scan	Grossly large mass within the body of the thymus without evidence of invasion. Tissue samples consisted of thyroid tissue closely associated with atrophic thymic tissue.
^5^	55	Metastatic papillary thyroid carcinoma	Palpable mass	No thymic / mediastinal imaging.	Neck dissection specimen contained involuting thymic tissue containing Hassall’s corpuscles. Within the thymic capsule were multiple cystically dilated thyroid follicles filled with colloidal fluid. The lining epithelium of the follicles was composed of flat cuboidal cells. The nuclei were uniformly small and ovoid. Intranuclear grooves or pseudoinclusions were not present.
^6^	55	Visual disturbance and intermittent chest pain	Normal	Large cervical goiter and a solid contrast-enhancing mass in the left anterior mediastinum contiguous to the thymus.	Grossly, large mass measuring 9 × 7.5 × 6.5 cm was found in the left inferior pole of the thymus, which had no direct physical continuity with the cervical goiter. Histopathologically, the thymus contained a large, well-encapsulated irregular mass with multiple small cysts consisting of multinodular thyroid tissue in an otherwise normal thymus.
^8^	37	History of breast malignancy and concern for metastasis	Palpable mass	CT scan revealed a large calcified anterior mediastinal mass distinct from the existing cervical goiter.	Grossly, a large anterosuperior mediastinal, lobulated mass, firm in consistency separate from cervical goiter. Histologically, mature thyroid tissue composed of thyroid follicles containing colloids of varying sizes, surrounded by thymic tissue.

In our patient and other reported cases in the literature, the morphology was that of normal follicular thyroid tissue surrounded by unremarkable thymic tissue. Obvious, abundant colloid with normal epithelium is a common reference point in the literature for these cases that was indicative of normal and benign thyroid gland tissue presence. Hwang et al.^5^ utilized galectin-3, HBME-1 and RET oncoprotein in addition to a lower Ki-67 labeling index to confirm that the heterotopic thyroid tissue was benign rather than neoplastic tissue (metastatic thyroid carcinoma).

While there have been documented cases of ectopic thyroid tissue transforming to a Graves-like goiter, it is not understood why some do or do not undergo transformation.^10^ In the case of our patient, the mass was hypertrophied enough to be felt on physical exam. However, on histology, it is extremely challenging to differentiate diffuse goiter of Graves disease from normal thyroid tissue, especially in the setting of medically treated Graves’ disease.

Our patient and others reported in the literature were treated by resection shortly after the imaging workup. The prognosis is good, with a limited chance of recurrence.
